# The Use of Donor-Derived Cell-Free DNA for Assessment of Allograft Rejection and Injury Status

**DOI:** 10.3390/jcm9051480

**Published:** 2020-05-14

**Authors:** Charat Thongprayoon, Pradeep Vaitla, Iasmina M. Craici, Napat Leeaphorn, Panupong Hansrivijit, Sohail Abdul Salim, Tarun Bathini, Franco H. Cabeza Rivera, Wisit Cheungpasitporn

**Affiliations:** 1 Division of Nephrology, Department of Medicine, Mayo Clinic, Rochester, MN 55905, USA; charat.thongprayoon@gmail.com (C.T.); craici.iasmina@mayo.edu (I.M.C.); 2 Division of Nephrology, Department of Medicine, University of Mississippi Medical Center, Jackson, MS 39216, USA; pvaitla@umc.edu (P.V.); sohail3553@gmail.com (S.A.S.); fcabezarivera@umc.edu (F.H.C.R.); 3 Renal Transplant Program, University of Missouri-Kansas City School of Medicine/Saint Luke’s Health System, Kansas City, MO 64111, USA; napat.leeaphorn@gmail.com; 4 Department of Internal Medicine, University of Pittsburgh Medical Center Pinnacle, Harrisburg, PA 17105, USA; hansrivijitp@upmc.edu; 5 Department of Internal Medicine, University of Arizona, Tucson, AZ 85724, USA; tarunjacobb@gmail.com

**Keywords:** donor derived cell free DNA, donor-derived cell-free DNA, ddcfDNA, cfDNA, kidney transplantation, renal transplantation, transplantation, kidney, nephrology, biomarkers

## Abstract

Patient monitoring after kidney transplantation (KT) for early detection of allograft rejection remains key in preventing allograft loss. Serum creatinine has poor predictive value to detect ongoing active rejection as its increase is not sensitive, nor specific for acute renal allograft rejection. Diagnosis of acute rejection requires allograft biopsy and histological assessment, which can be logistically challenging in some cases and carries inherent risk for complications related to procedure. Donor-derived cell-free DNA (dd-cfDNA), DNA of donor origin in the blood of KT recipient arising from cells undergoing injury and death, has been examined as a potential surrogate marker for allograft rejection. A rise in dd-cfDNA levels precedes changes in serum creatinine allows early detections and use as a screening tool for allograft rejection. In addition, when used in conjunction with donor-specific antibodies (DSA), it increases the pre-biopsy probability of antibody-mediated rejection (ABMR) aiding the decision-making process. Advancements in noninvasive biomarker assays such as dd-cfDNA may offer the opportunity to improve and expand the spectrum of available diagnostic tools to monitor and detect risk for rejection and positively impact outcomes for KT recipients. In this this article, we discussed the evolution of dd-cfDNA assays and recent evidence of assessment of allograft rejection and injury status of KT by the use of dd-cfDNA.

## 1. Introduction

Kidney Transplantation (KT) is the best treatment option for patients with end-stage kidney disease (ESKD) [1]. It provides better patient survival, especially a marked decrease in cardiovascular mortality when compared to maintenance dialysis [2]. However, allograft loss remains a major issue for KT patients [3]. While there has been improvement in one-year graft survival and allograft rejection, there is little improvement in the long-term rate of graft loss [4,5]. Current KT surveillance options for allograft injury such as serum creatinine (SCr), urinalysis, urinary protein, donor specific antibody (DSA), and BK virus surveillance have known limitations [6,7,8]. Transplant providers have encountered the challenge to identify allograft rejection using non-sensitive biomarkers and clinical signs/symptoms.

Although SCr or eGFR remains the mainstay for assessment of renal allograft function, monitoring the trends of SCr has poor predictive value to detect active rejection. An increase in SCr is not sensitive, nor specific to acute rejection of a kidney allograft. Furthermore, it is also a late signal. Approximately 17% of transplant centers in the United States perform surveillance KT biopsies [9]. While recent study demonstrated that the one- and three-year observed expected graft survivals are comparable among centers performing surveillance biopsies vs. those not performing biopsies [9], several studies have shown important values of surveillance KT biopsy on predictions of allograft loss [10,11]. Although KT biopsy is the gold standard to identify allograft dysfunction, it is an invasive procedure, not without complications, and can encounter challenges including sampling errors, inadequate tissue sample, and variability of interpretation among pathologists [12,13]. 

Thus, an urgent need exists for noninvasive and sensitive diagnostic tools for the detection of early rejection in KT that precedes a rise in SCr, and offers the opportunity to better inform therapeutic decision making [14,15]. In non-KT patients, the utilizations of novel acute kidney injury (AKI) biomarkers—neutrophil gelatinase-associated lipocalin (NGAL) and kidney injury molecule-1 (KIM-1)—may help predict AKI prior to the rise of SCr [16]. However, these novel AKI biomarkers are more reflective of ischemic rather than alloimmune graft injury in KT population, and are not associated with post-KT graft outcomes at a median four years post-KT [17]. 

For the past decade, the development of novel technologies (Table 1) applied to the monitoring of acute allograft rejection include genomics, transcriptomics, proteomics, and metabolomics, which quantify the abundance of circulating cell free DNA, gene transcripts (mRNA), proteins, and metabolites, respectively, in cell/tissue extracts or biofluids [14,15,18,19,20,21,22,23,24,25,26,27,28,29,30,31,32,33,34,35,36,37,38,39,40,41,42]. These technologies have advanced the non-invasive diagnosis of acute rejection among KT patients and allow early identification of allograft injury and timely intervention. Currently, genomic-based assays that measure donor-derived cell-free DNA (dd-cfDNA) in the serum have qualified for Medicare coverage. Other assay technologies that measure gene transcripts (mRNA), proteins, and metabolites are active areas of research. A commercialized plasma/blood transcriptomic assay has also qualified for Medicare coverage. 

## 2. The Evolution of Donor-Derived Cell-Free DNA Assays 

An emerging area of research has been the advent of assays that detect donor-derived cell-free DNA (dd-cfDNA) [14,23,43]. dd-cfDNA, DNA of donor origin in the blood of the KT recipient, has developed as a noninvasive marker suggestive of allograft rejection, since it originates from cells undergoing injury and death, and can be found in serum, plasma, urine, saliva, feces, synovial fluid, CSF, and peritoneal fluid. cfDNA technology has been utilized in prenatal testing and oncology, and research for the past decade has led to the application and development of this technology for evaluation of allograft rejection [44,45].

Following organ transplantation, dd-cfDNA circulates in the recipient’s blood, and accounts for a relatively small fraction of total cfDNA (recipient plus donor derived) [46]. As cells from the donor allograft degrade, the nucleic acids within become fragmented, resulting in approximately 120–160 base pair pieces of double-stranded dd-cfDNA released into the blood, and cleared from blood by the liver and kidney with half-life of about 30 min [47]. The mechanisms of release of cfDNA into the bloodstream is believed to be a result of several possible mechanisms, including cell death by apoptosis or necrosis, in addition to active secretion by various activated cells of the immune system [48,49,50,51,52]. 

**Figure 1:**
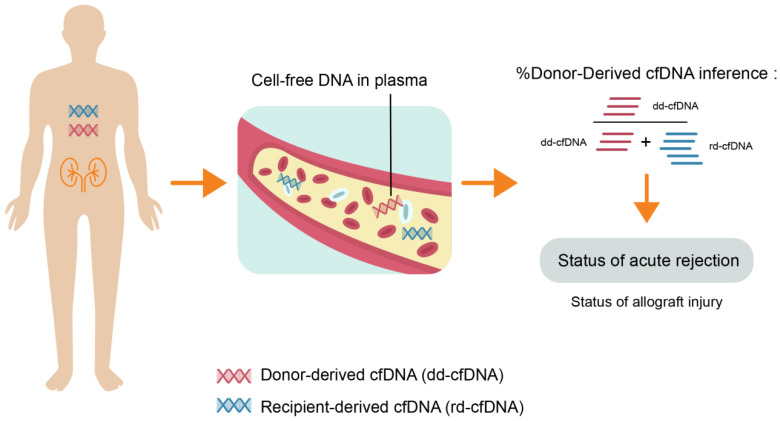
The Use of dd-cfDNA Assays to Assess Status of Acute Rejection and Allograft Injury.

The test measures the proportion of total cell-free DNA that is derived from the donor and the recipient (Figure 1). dd-cfDNA is typically low in concentration, only a few thousand genomic copies/mL [53], and dd-cfDNA is usually <1% of the total cell-free DNA when there is no active damage to the allograft [46]. However, during allograft rejection, significantly higher amounts of dd-cfDNA are released from the injured allograft into the bloodstream [46,47]. Early rises of total dd-cfDNA levels during acute rejection have been observed in KT recipients [48,54]. These observations supported the premise for quantitative measurement and interpretation of dd-cfDNA as a tool to evaluate the relative health of a KT allograft and diagnose possible complications such as acute allograft rejection [45,46,53,55,56]. 

The measurement of dd-cfDNA in a transplant recipient involves blood being drawn into specialized tubes that preserve nucleic acids, followed by plasma isolation by centrifugation. The presence of cell free DNA in the circulation was first described over six decades ago by Mandel and Metais in 1948 three years before the double helix discovery [57]. In transplant medicine, the presence of donor-derived DNA in the recipient’s plasma (or urine), called microchimerism, has been known since the end of 1990s and its use as a measure of transplant injury has been tested [58]. Early technologies to differentiate donor and recipient genomes to specifically quantify dd-cfDNA in transplant recipients required either gender mismatch between donor and recipient or prior genotyping of the donor and recipient. These techniques were not widely used due to practical limitations and additional costs [44,59].

More recently, dd-cfDNA can be extracted from plasma samples and used for polymerase chain reaction (PCR) amplification, sequencing and analysis. Methods for measuring dd-cfDNA include quantitative reverse transcription PCR (RT-PCR), droplet digital PCR (ddPCR), and targeted next-generation sequencing [23]. Recent advances in PCR and next generation sequencing (NGS) have made its detection feasible and cost effective [46]. Next-generation sequencing assays use polymorphisms between donor and recipient to discriminate dd-cfDNA from recipient-cfDNA. These techniques allow targeted amplification and sequencing of single-nucleotide polymorphisms (SNP) to quantify donor and recipient DNA contributions, without the need for prior genotyping of the donor and recipient [44]. 

## 3. dd-cfDNA and Renal Allograft Rejection

Detection of dd-cfDNA in the blood circulation is an early marker of injury in solid organ transplantation [45,60,61,62]. The biological rationale for the utility of dd-cfDNA in KTx is that cell damage to the allograft leading up to or during episodes of rejection results in release of DNA in the circulation of the recipient and therefore an uptick in the dd-cfDNA levels [45,55]. In stable KT patients, the level of plasma dd-cfDNA is usually <1%, with one study showing median of 0.21% [45]. A serial change in the level of dd-cfDNA to more than 61% from the first value was considered outside the expected biological variation [55]. Baseline levels of dd-cfDNA are higher in deceased donor vs living donor recipients indicating ischemic reperfusion injury. Levels increase immediately post KT and decrease to about <1% by two weeks post-transplant. After two weeks post-KT, a subsequent increase can indicate renal allograft injury. dd-cfDNA decreases in response to treatment of rejection [53]. Its role in allograft rejection is being actively investigated in many solid organ transplants including kidney, heart, lung and liver [44]. 

In a multicenter study, Bloom et al., was the first to report the use of dd-cfDNA in KT recipients measured by the targeted next-generation sequencing assay to detect active allograft rejection [45]. In 102 patients with 107 for-cause biopsies correlated with blood samples for dd-cfDNA, 27 had rejection episodes. The median dd-cfDNA levels were lower among controls (without histological rejection) (0.3%), when compared to patients with antibody-mediated rejection (ABMR)) (2.9%), T cell-mediated rejection (TCMR) grade ≥IB (1.2%), and TCMR grade IA (0.2%) [45]. A cutoff point of dd-cfDNA levels >1% indicated a probability of active rejection with the receiver operating characteristic area under the curve (AUROC) of 0.74. However, this assay was elevated in TCMR grade >1B but not TCMR grade 1A. The study concluded that dd-cfDNA may be used to assess allograft injury and a level <1% reflected an absence of active rejection (TCMR >IB or ABMR) [45]. However, a limitation of this study included a small number of protocol biopsies. These reported test characteristics therefore do not apply to detecting subclinical rejection (SCR) in patients with stable allograft function. Negative results are strongly supportive of no active injury, high negative predictive value (NPV), and using dd-cfDNA level of 0.2% provided 95% NPV for acute renal allograft rejection. 

Additional studies have shown mixed results. Huang et al. [63] found that for ABMR, the AUC was 0.82 (95% CI: 0.71-0.93) and a dd-cfDNA ≥ 0.74% yielded a sensitivity of 100%, and specificity of 71.8%. However, dd-cfDNA test did not discriminate cellular rejection from no rejection among KT recipients [63]. Gielis et al. [64] assessed 107 KT recipients and collected blood for dd-cfDNA at 10 timepoints over the first three months after KT, at times of SCr rise, and protocol or for cause biopsy. Increases in dd-cfDNA were associated with episodes of acute rejection, acute tubular necrosis, and acute pyelonephritis. However, it performed no better than SCr in diagnosing acute rejection. In another analysis of dd-cfDNA with paired DSA samples in clinically indicated biopsies, Jordan et al. [61] demonstrated that in DSA positive patients, dd-cfDNA performed better for diagnosis of ABMR. The study concluded that patients with dd-cfDNA+/DSA+ results have high probability of active ABMR. dd-cfDNA > 2.9% is highly specific in distinguishing ABMR from no ABMR with an 89% positive predictive value (PPV). 

Recently, Sigdel et al. [65], in a single-center retrospective study, reported a different dd-cfDNA method that uses a NGS assay with single nucleotide polymorphisms (SNP)-based massively multiplex polymerase chain reaction (mmPCR). The investigators looked at 300 plasma samples collected from 193 KT patients including those receiving protocol biopsies. They included 217 biopsy-matched: 38 plasma samples from patients with active rejection, 72 borderline TCMR rejection, 82 samples from patients with stable allografts, and 25 samples from patients with other injury collected from193 KT patients including those receiving protocol biopsies. dd-cfDNA was processed by mmPCR targeting 13,392 SNPs. With a specified cutoff of 1%, the test was able to discriminate acute allograft rejection (both ABMR and TCMR) from non-rejection with an AUROC curve of 0.87 (88.7% sensitivity, 72.6% specificity, NPV 95.1%, and PPV of 51.9%). Unlike the other dd-cfDNA technology, the test was able to distinguish TCMR and ABMR from no rejection. The technical advances made possible a highly sophisticated approach of mmPCR allowing the utilization of more than 13,000 SNP markers [65]. The results were similar for protocol biopsies vs for-cause biopsy samples, living vs deceased donors and importantly, showed the ability to distinguish both ABMR and TCMR cases (>1A) from borderline TCMR or other injuries including toxic injury or viral infection. There are several important limitations in this study [65]. In addition to the retrospective nature of the study in a single institution, there are several points to be noted. The acute rejection group contained mostly for-cause biopsies whereas the non-rejection group contained mostly surveillance biopsies. This is important because the incidence of rejection is known to be significantly greater in for-cause biopsies. Nevertheless, a study by Altuğ et al., that included six transplant patients confirmed the assay’s analytical validity and performance with respect to detecting acute rejection in KT recipients, regardless of donor–recipient relationships [56].

Among KT recipients with retransplantation, recent study of 12 repeat kidney transplant recipients with retained allografts and 202 single KT recipients showed that the median dd-cfDNA levels were significantly lower than threshold levels for rejection in both single kidney transplant recipients and repeat kidney transplant recipients [66]. The findings of this study suggested that dd-cfDNA can be utilized to evaluate renal allograft status in repeat transplant recipients. 

## 4. Potential Directions and Future Scope 

In addition to its use for acute renal allograft rejection, recent evidence suggests potential use of dd-cfDNA for monitoring and assessment of injury status of renal allograft [45,55,65,67,68]. dd-cfDNA is elevated in the presence of allograft injury, and portends adverse posttransplant events such as eGFR decline, formation of de-novo donor specific antibodies and allograft rejection across many types of solid organ transplants [69,70,71]. Among KT recipients, dd-cfDNA levels are elevated at the time of acute rejection and decline over a period of three months to near reference levels, which may confirm real-time response to treatment of acute rejection [72]. Thus, the use of dd-cfDNA may be useful to detect and subsequently assess recovery from acute rejection and improve the long-term monitoring of KT recipients. A recent multicenter study examined early TCMR (borderline and Banff 1A) and demonstrated that recipients with elevated levels of dd-cfDNA (>0.5%) were associated with adverse outcomes including decline in eGFR, de novo DSA formation as well as increased risk of future or persistent rejection compared to recipients with dd-cfDNA <0.5% [67]. Emerging evidence also suggests that the elevation of dd-cfDNA precedes the development of de-novo DSAs (including non-HLA DSAs) and eGFR decline [67,69]. Interestingly, recent evidence has suggested that dd-cfDNA may itself be a trigger of inflammation, thereby adding insult to injury [62]. 

Considering dd-cfDNA as a continuous and clinically significant biomarker opens up potential for new management strategies, therapeutics, and ways to quantify interventions exploring the immunological potential of dd-cg-DNA. Following levels of dd-cfDNA over time may provide windows of opportunity to intervene, for instance by augmenting immunosuppression to prevent acute rejection, prior to the occurrence of adverse events. This approach may enable clinicians to take a proactive rather than reactive approach to posttransplant patient management. dd-cfDNA is clearly a marker of allograft damage and rejection. In routine clinical settings both tests have been approved for monitoring patients with a functional kidney transplant for rejection, and are reimbursed by Medicare. Whether and how best to incorporate dd-cfDNA as a non-invasive marker in the care of patients following transplantation remains an area of active debate. Advantage of dd-cfDNA lies in its high NPV, which in the absence of DSA further reduces the probability of ABMR diagnosis. However, its ability to accurately predict low-grade TCMR should be further evaluated. Furthermore, like every diagnostic test, the result has to be interpreted in the right context and its limitations understood. Since dd-cfDNA increases with allograft injury, the effects of BK nephropathy, glomerulonephritis or active urinary tract infection on its level need further evaluation. At this time, its use in patients with multiple-organ transplants is not recommended. 

Studies directly comparing different dd-cfDNA determination methods are lacking. It is encouraging, however, that the median values of clinically stable patients were similar in different studies using different methods for dd-cfDNA determination [45,55,65,68]. Assays which detect dd-cfDNA, differ in technology and approach, sometimes significantly, and therefore each new assay requires clinical validation before applicability can be assessed. Future studies should include a large cohort of cases with different diagnoses, as well as assess effect on clinical outcomes of treatment strategies based on utilizing dd-cfDNA versus traditional methods. The ongoing study titled ‘Evaluation of Patient Outcomes from the Kidney Allograft Outcomes AlloSure Registry (KOAR)’ would definitely serve this purpose. The advent of dd-cfDNA assays is a highly promising opportunity to detect rejection early and noninvasively. This would offer several benefits, such as optimizing biopsy use (e.g., more targeted biopsies) and improving immunosuppression use (e.g., monitoring after immunosuppression adjustments or confirming patient adherence), thus providing opportunities to improve graft survival rates. Clinical trials are ongoing to further study the efficacy and utility of dd-cfDNA assays to detect allograft rejection and their ability to positively impact outcomes in kidney transplantation (NCT03765203, NCT03984747, NCT04091984, and NCT03759535).

## 5. Conclusions

Acute allograft rejection remains an important problem in KT, causing adverse impacts on allograft outcomes. Advancements in specific noninvasive biomarker assays, such as dd-cfDNA, provide the opportunity to improve and expand the spectrum of available diagnostic tools to monitor and detect risk for rejection and positively impact outcomes for KT recipients. Future multicenter studies for the use of dd-cfDNA for non-invasive longitudinal monitoring of injury status of renal allograft correlated with histological assessment are needed.

## Figures and Tables

**Table 1:** Non-Invasive Diagnosis and Prognostication of Acute Allograft Rejection Kidney Transplant Recipients.

Non-Invasive Diagnosis and Prognostication of Acute Allograft Rejection Kidney Transplant Recipients
- Donor-derived cell-free DNA (dd-cfDNA) - Blood gene expression profiles (Trugraf, kSORT) - Urinary mRNA (e.g., perforin, granzyme B, IFN-inducible protein-10 [IP-10], CD3ε mRNA) - Urinary levels of chemokine (CXCL9 and CXCL10) - Proteomic and peptidomic signatures of acute rejection in urine and blood samples - IFN-gamma enzyme-linked immunospot (ELISPOT) assay

Abbreviations: kSORT, the Kidney Solid Organ Response Test; IFN, Interferon; CXCL9; CXCL10; ELISPOT, enzyme-linked immunospot assay.
